# The Clinical Outcomes of COVID-19 in Patients with Behçet’s Disease: A Series of 7 Cases and Brief Review of the Literature

**DOI:** 10.5152/eurasianjmed.2021.21203

**Published:** 2022-10-01

**Authors:** Kemal Nas, Gamze Dilek, Mehtap Kalçık Unan, İbrahim Tekeoğlu, Ayhan Kamanlı

**Affiliations:** 1 Division of Rheumatology and Immunology, Department of Physical Medicine and Rehabilitation, Sakarya University School of Medicine, Sakarya, Turkey

**Keywords:** Behçet’s disease, COVID-19, management, colchicine, clinical course

## Abstract

Conventional debates surrounding the treatment of coronavirus disease 2019 still continue in the literature. Colchicine is one of the recommended agents in the treatment of coronavirus disease 2019, but there are also studies giving negative opinions for the said agent. Some researchers suggest that those patients using colchicine have milder clinic symptoms.

Behçet’s disease is a multisystemic disease with an uncertain etiopathogenesis that is characterized by chronic inflammatory vasculitis. Autoimmunity is believed to play a key role in the pathogenesis of Behçet’s disease. Immunomodulator, corticosteroid, and immunosuppressive drugs are used in its treatment.

Seven Behçet’s patients with coronavirus disease 2019 were presented in this study, and the drugs used, prognosis, accompanying diseases, hospitalization, and complications were discussed in the light of the literature.

**Main Points**

- We believe that our Behçet’s disease patients are no more susceptible to Coronavirus disease 2019 (COVID-19) than the normal population.
- We suggested that immunosuppressive and immunomodulatory treatments of Behçet’s patients should not be discontinued during the COVID-19 period.
- We recommended that additional comorbid diseases of Behçet’s patients should be well managed during the COVID-19 period.’

## Introduction

Coronavirus disease 2019 (COVID-19) has required a global fight since December 2019. Patients with underlying medical conditions such as rheumatic autoimmune diseases and vasculitis face a higher risk of COVID-19 and associated complications.

Even though there are many studies investigating the characteristics of the computed tomography (CT) findings of COVID-19, there is only limited research analyzing the imaging characteristics in patients with rheumatic diseases.^1–4^

It is also known that rheumatic diseases can cause damage to the tissues, secondary to inflammation, through a similar mechanism. Radiological findings of tissue damage that occurs as a result of this pathological inflammatory process related to the current rheumatic disease might overlap with the radiological findings of COVID-19.^1^ Pulmonary parenchymal manifestations of Behçet’s disease include interstitial lung disease, pulmonary nodules, and bronchiectasis.^5^ In assessing interstitial involvement of COVID-19 in patients with accompanying Behçet’s disease, processes in both diseases should be taken into consideration.

Behçet’s disease (BD) represents a form of vasculitis that mostly involves the venous system. It can involve arteries and veins of any size.^6^ In general, vascular involvement occurs during the first 5 years of the disease. There is an underlying pathological process of neutrophil infiltration and vascular inflammation in the perivascular area.^6^ Coronavirus disease 2019 can damage the immune system and cause release of large amounts of cytokines, which is called a cytokine storm.^4^

Colchicine is an anti-inflammatory agent that inhibits the polymerization of cellular microtubules and the formation of NLRP3 inflammasome.^7^

In this study, pre- and post-clinical data, radiological and laboratory findings, and treatment prognosis of 7 BD patients that contracted COVID-19 are presented in a systematic literature review (Table 1).

## Case Presentations

### Case 1

A 47-year-old female patient was followed up with a BD diagnosis for 10 years. The patient was on a colchicine regimen of 1 mg/day and an azathioprine regimen of 50 mg/day. She had no comorbidities. She presented to the emergency room with severe back pain and loss of taste and smell. A combined nasal and throat specimen produced a positive polymerase chain reaction (PCR) test result. A regimen of 2 × 1600 mg of favipiravir was administered on the first day as the loading dose, to be followed by 2 × 600 mg on the remaining 4 days as the maintenance dose. One month after complete recovery, an erythema nodosum lesion occurred on the front of the right leg. She did not experience Behçet’s related exacerbations after COVID-19.

### Case 2

A 48-year-old female patient was followed up for 5 years without any medication. The patient suffered from oral aphthous ulcers every 3-4 months. She did not have any comorbidities. She presented to the emergency room with complaints of dyspnea, nausea, vomiting, arthralgia, myalgia, diarrhea, and loss of taste and smell. A combined nasal and throat specimen produced a positive PCR result. She was hospitalized with bilateral lung involvement and stayed at the hospital for 3 days. She was administered antibiotics, analgesics and favipiravir, in the form of 2 × 1.600 mg of loading dose on the first day and 2 × 600 mg of maintenance dose on the remaining 4 days. An enoxaparin regimen of 6000 IU/0.6 mL 1 × 1/day was also administered. She was discharged without experiencing any complications. As she suffered increased oral aphthous ulcers after COVID-19, a colchicine regimen of 2 × 1/day was started.

### Case 3

A 52-year-old female patient was followed up with BD diagnosis for 20 years. The patient was being treated with 1 mg of colchicine, 50 mg 1 × 1/day of azathioprine, and 2 mg/day of methylprednisolone. She had iatrogenic hypocalcemia. She presented to the emergency room with complaints of severe muscle and joint pain, diarrhea, and dyspnea. She was hospitalized as her combined throat and nose specimen produced a positive PCR result. Her C-reactive protein was found to be 104, whereas other parameters were within normal ranges. Bilateral patches of ground-glass opacities were detected in the patient’s thoracic CT. She was hospitalized for 5 days to receive 2 × 400 mg of hydroxychloroquine on the first day, to be followed by 2 × 200 mg on the remaining 4 days, as well as 2 × 1.600 milligrams of favipiravir on the first day, to be followed by 2 × 600 mg on the remaining 4 days, and 4000 IU 1 × 1/day enoxaparin, corticosteroids, and antibiotics. There were no changes in her Behçet’s-related symptoms after COVID-19.

### Cases 4 and 5

These are patients that presented to the hospital with complaints of myalgia, fever, cough, nausea, and loss of taste and smell. Combined nasal and throat specimens produced a positive PCR test result in both patients. Neither of the patients had comorbidities. They were on a colchicine regimen of 1.5 mg/day. For COVID-19, they received 2 × 1.600 mg of favipiravir on the first day, to be followed by 2 × 600 mg on the remaining 4 days. Their laboratory results were within normal ranges. They had full remission. There were no changes in their Behçet’s related symptoms after COVID-19.

### Case 6

A 57-year-old male patient was followed up with BD diagnosis for 35 years. The patient was on a colchicine regimen of 1.5 mg/day and methylprednisolon regimen of 4 mg/day. He had a history of surgery for pancreatic cancer. He had bilateral vision loss due to Behçet’s disease. He presented to the emergency room with complaints of fever and malaise. His combined throat and nose swab gave a positive PCR test result. He was admitted to the hospital. (Laboratory parameters of the patient were LDH 217 U/L, ferritin 75 ml/ng, D-dimer 317 ng/ml). His oxygen saturation was around 95. Bilateral patches of ground-glass opacities were detected in the thoracic CT. He received 2 × 1.600 mg of favipiravir on the first day, to be followed by 2 × 600 mg on the remaining 4 days, as well as enoxaparin of 6000 IU/0, 6 mL 1 × 1/day. After 7 days, he was discharged without experiencing any complications. After COVID-19, he suffered more frequent stomachache complaints.

### Case 7

A 51-year-old male patient was followed up with BD diagnosis for 20 years. The patient was on a colchicine regimen of 1.5 mg/day. He had accompanying epilepsy and food allergies. He presented to the emergency room with complaints of loss of taste and smell and back pain. His combined throat and nose swab gave a positive PCR test result. His laboratory parameters were within normal ranges. He was hospitalized and put under observation as bilateral patches of ground-glass opacities were detected in his thoracic CT. He received a favipiravir regimen of 2 × 1.600 mg on the first day, to be followed by 2 × 600 mg on the remaining 4 days. He was discharged without experiencing any complications. He did not have Behçet’s related exacerbations after COVID-19.

All the patients gave their written consent to the study.

## Discussion

Behçet’s disease is a rare idiopathic inflammatory multisystem disease. Uveitis and recurrent oral and genital aphthous ulcers represent the typical triad of this disease.^8^ Even though veins are frequently involved, both arteries and veins can be affected in the form of vasculitis.^9^ Unlike other rheumatic diseases, BD is common only in a few countries that are mostly located along the ancient Silk Road.

In this case series, we aimed to present therapeutic practices around a common disease in our country, namely Behçet’s disease, during the pandemic period by combining observations of rheumatology physicians monitoring BD patients with COVID-19 and our clinical experience. Clinical, radiological, and laboratory findings should be evaluated in BD patients with COVID-19 considering the differential diagnosis of pulmonary and vascular involvement. Unfortunately, there are only a few published studies on BD patients with COVID-19.

In this study, we reviewed 7 patients with a positive PCR result for COVID-19. Three of the patients had comorbidities. The patient with a total vision loss caused by Behçet’s disease had the longest hospital stay. In our case series, 1 hospitalized patient who was not on colchicine experienced an increased frequency of oral aphthous ulcers. This patient was clinically stable and received follow-up care; however, she was re-prescribed colchicine upon worsening of her oral ulcer complaints after COVID-19.

In a study conducted by Berna et al.^10^ there was an increase in symptoms that could be associated with BD in a case series of 10 BD patients with COVID-19. In this study, 1 of the patients had deep vein thrombosis (DVT) and 2 of them had an increased frequency of oral aphthous ulcers.

During the course of COVID-19, proinflammatory cytokines and neutrophil extracellular traps, which are involved in the etiopathogenesis of vasculitides affecting small vessels, can also be associated with the infection, inflammation, and thrombosis in COVID-19 pathogenesis.^11^ Furthermore, BD initiates neutrophil hyperfunction and the coagulation cascade via tissue factor activation.^12^ COVID-19 can also damage the endothelium and cause hypercoagulability. A higher risk of arterial and venous thrombosis is reported during the course of COVID-19.^13^ Considering the similar pathways of vascular inflammation, there might be an increased risk of thrombosis in both diseases. However, there is no sufficient data in the literature to base this theory on. In our series, we did not detect any thrombosis.

In Turkey, COVID-19 treatment algorithms drafted by the Ministry of Health are updated regularly. In the absence of contraindications, the current algorithms recommend low molecular weight heparin treatment in adults with COVID-19 at prophylactic doses. In line with these algorithms, patients in our series were hospitalized in COVID-19 inspection ward and administered anticoagulant therapy.^14^

A study by Berna et al^10^ reports DVT in one of the BD patients with COVID-19. In this patient, immunosuppressive therapy might have been paused to manage the risk of COVID-19, which might have caused BD-related exacerbations and increased risk of thrombosis.

In the literature, it is reported that colchicine could be used for the treatment of patients with COVID-19 given its anti-inflammatory features. Neutrophil-related inflammasome formation plays a major role in the pathogenesis of acute respiratory distress syndrome (ARDS). In patients with COVID-19, ARDS is a common clinical complication.^15^ However, in randomized studies, it was found that colistin treatment during COVID-19 was not effective in reducing hospitalization, mechanical ventilation, and mortality rate in the general population.^16,17^ International Society for Behçet’s Disease (ISBD) has advised that not to discontinue Colchicine to prevent a relapse of the disease in BD patients with COVID-19.^18^

On the other hand, a meta-analysis reviewing therapeutic recommendations for BD patients during the COVID-19 pandemic concludes that colchicine, which might positively impact the prognosis, should be used during COVID-19. Studies in the meta-analysis suggest that conventional immunosuppressive therapies and biological agents should continue to be administered during the pandemic but may as well be withdrawn upon the physician’s decision.^19,20^

Given the literature data, we are convinced that while colchicine decreases neutrophil migration, it can also decrease the level of inflammation in BD patients with COVID-19.

In our series, the patient with the longest hospital stay was the one with the permanent vision loss caused by BD who had a history of pancreatic cancer surgery. This is in line with the reports in the literature that COVID-19 patients with multiple comorbidities have a higher hospitalization rate.^21,22^

We believe that our BD patients are no more susceptible to COVID-19 than the normal population, but 2 patients are required to support in the hospital setting (antibiotics and steroids) because of their comorbidities (pancreatic ca, epilepsy, etc.).

Our patients were subjected to routine follow-up every 3 months in the rheumatology polyclinic. However, after they contracted COVID-19, their routine follow-up frequency was reduced to every month for the next 3 months.

In our case series, a BD patient that had no accompanying disease or a colchicine history was hospitalized with COVID-19 and experienced an increased frequency of oral ulcers. Such clinical activation was not seen in other patients. Colchicine is commonly used to prevent autoinflammatory diseases.^23^ In an Familial Mediterranean Fever (FMF) case series, it is reported that colchicine might prevent cytokine storms and could be used in the treatment of COVID-19 patients due to its inhibitory effects on intracellular cytokine and chemokine secretion.^24^ In a study conducted by Espinosa et al.^25^ 4 female COVID-19 patients were on colchicine. Two of these patients with mucosal, ocular, and vascular involvement receiving MTX therapy were hospitalized. These 2 patients exhibited COVID-19 symptoms for a longer time compared with the other 2 patients on colchicine. In our study, 2 patients who were on colchicine were both hospitalized. They had a longer disease duration, bilateral lung involvement, and methylprednisolone therapy in common. In the literature, corticosteroid use (over 3 months and more than 10 mg) is associated with a poor prognosis during COVID-19.^21^ Our patients were on a low dose (4 mg) of methylprednisolone; however, prolonged use may have weakened their immune system. Besides, not being on colchicine might have triggered mucosal inflammation associated with BD after COVID-19.

In this study, we also evaluated the treatment practices of rheumatologists during and after the disease in BD patients with COVID-19.

## Conclusion

In a series of 7 BD patients with COVID-19, we tried to analyze the causes of symptoms and findings taking the literature data into consideration. We believe that use of colchicine may be effective in the treatment of COVID-19 patients, and the use of colchicine in BD patients may have a role in a milder clinical presentation of COVID-19. However, considering the prevalence of BD around the world, reported cases are only a few. Including higher number of cases in such studies will reduce the said limitation and help obtain a clearer picture.

## Figures and Tables

**Table 1.** Clinical and Demographic Characteristics of Behcet’s Patients in Our Study and Literature

**Article**	**Age/Sex**	**Type of Study**	**Disease/Severity**	**Comorbitdity**	**Medical History**	**Imaging Findings**	**COVID-19 Symptoms at Presentation**	**COVID-19 Course**
**Yurttaş B et al. 10**	5F (mean age 34,4) 5M (mean age 41,2)	Case series 10 BD	1 Patient died 2 Patients required ICU6 Patients had pneumonia8 Patients were hospitalized	1 Epilepsy 1 Anti-TNF-induced psoriasis1 Endometrium cancer1 Psychiatric disease	Colchicine Azathioprine Methylprednisolone	Interstitial pneumonia in CT scan in 3 patients	6 Fever 6 Cough5 Myalgia1 Anosmia2 Arthralgia1 Asphyxia	HCQ Oseltamivir Azathioprine Favipiravir Enoxaparine Methylprednisolone
**Espinosa G et al. 19**	5F (mean age 43,75)	Case series 4 BD	3 Patients were hospitalized	1 Breast cancer	Colchicine, MTX Azathioprine Methylprednisolone	Left basal ground-glass opacity in one patient	4 Cough 1 Anosmia1 Diarrhea3 Headache	Lopinavir/ritonavir HCQAzithromycin Methylprednisolone
**In this study**								
**Case 1**	47/F		Mild symptoms	None	Colchicine	NR	Loss of taste and smell Myalgia	Favipiravir
**Case 2**	48/F		Moderate symptoms / hospitalized	None	None	Interstitial pneumonia in CT scan	Dyspnea loss of taste and smell ArthralgiaMyalgiaNausea	Favipiravir Enoxaparine Azithromycin
**Case 3**	52/K		Moderate symptoms/hospitalized	Hypocalcemia	Colchicine Azathioprine Methylprednisolone	Interstitial pneumonia in CT scan	Dyspnea Arthralgia Diarrhea Myalgia	Favipiravir Enoxaparine Ceftriaxone Methylprednisolon HCQ
**Case 4**	33/F		Mild symptoms	None	Colchicine	NR	Fever Headache Arthralgia Myalgia Cough loss of taste and smell	Favipiravir
**Case 4**	33/F		Mild symptoms	None	Colchicine	NR	Fever Headache Arthralgia Myalgia Cough loss of taste and smell	Favipiravir
**Case 5**	41/K		Mild/moderate symptoms	None	Colchicine	Interstitial pneumonia in CT scan	Fever Headache Arthralgia Myalgia Dyspnea Diarrhea Loss of taste and smell	Favipiravir
**Case 6**	57/M		Moderate symptoms/ hospitalized	Pancreatic cancer, Depression	Colchicine Methylprednisolone	Interstitial pneumonia in CT scan	Fatigue	Favipiravir Enoxaparine Azithromycin
**Case 7**	51/M		Moderate symptoms/ hospitalized	Epilepsy	Colchicine	Interstitial pneumonia in CT scan	Arthralgia Myalgia Loss of taste and smell	Favipiravir Azithromycin

BD; Behçet’s patients, MTX; methotrexate, ICU; intensive care unit, HCQ; hidroksiklorokin.
